# Beyond Traditional Advertisements: Leveraging Facebook’s Social Structures for Research Recruitment

**DOI:** 10.2196/jmir.3786

**Published:** 2014-10-27

**Authors:** Rupa S Valdez, Thomas M Guterbock, Morgan J Thompson, Jeremiah D Reilly, Hannah K Menefee, Maria S Bennici, Ishan C Williams, Deborah L Rexrode

**Affiliations:** ^1^Department of Public Health SciencesUniversity of VirginiaCharlottesville, VAUnited States; ^2^Center for Survey ResearchUniversity of VirginiaCharlottesville, VAUnited States; ^3^Department of SociologyUniversity of VirginiaCharlottesville, VAUnited States; ^4^Paris School of International AffairsInstitut d’Etudes Politiques de ParisParisFrance; ^5^School of NursingUniversity of VirginiaCharlottesville, VAUnited States

**Keywords:** participant recruitment, Facebook, social media, consumer health IT, ethnicity, advertising

## Abstract

**Background:**

Obtaining access to a demographically and geographically diverse sample for health-related research can be costly and time consuming. Previous studies have reported mixed results regarding the potential of using social media-based advertisements to overcome these challenges.

**Objective:**

Our aim was to develop and assess the feasibility, benefits, and challenges of recruiting for research studies related to consumer health information technology (IT) by leveraging the social structures embedded in the social networking platform, Facebook.

**Methods:**

Two recruitment strategies that involved direct communication with existing Facebook groups and pages were developed and implemented in two distinct populations. The first recruitment strategy involved posting a survey link directly to consenting groups and pages and was used to recruit Filipino-Americans to a study assessing the perceptions, use of, and preferences for consumer health IT. This study took place between August and December 2013. The second recruitment strategy targeted individuals with type 2 diabetes and involved creating a study-related Facebook group and asking administrators of other groups and pages to publicize our group to their members. Group members were then directly invited to participate in an online pre-study survey. This portion of a larger study to understand existing health management practices as a foundation for consumer health IT design took place between May and June 2014. In executing both recruitment strategies, efforts were made to establish trust and transparency. Recruitment rate, cost, content of interaction, and characteristics of the sample obtained were used to assess the recruitment methods.

**Results:**

The two recruitment methods yielded 87 and 79 complete responses, respectively. The first recruitment method yielded a rate of study completion proportionate to that of the rate of posts made, whereas recruitment successes of the second recruitment method seemed to follow directly from the actions of a subset of administrators. Excluding personnel time, the first recruitment method resulted in no direct costs, and the second recruitment method resulted in a total direct cost of US $118.17. Messages, posts, and comments received using both recruitment strategies reflected ten themes, including appreciation, assistance, clarification, concerns, encouragement, health information, interest, promotion, solicitations, and support. Both recruitment methods produced mixed results regarding sample representativeness with respect to characteristics such as gender, race, and ethnicity.

**Conclusions:**

The results of the study demonstrate that leveraging the social structures of Facebook for health-related research was feasible for obtaining small samples appropriate for qualitative research but not for obtaining large samples needed for quantitative research. The content of interactions with members of the target population prompted ethical deliberations concerning suitable target communities and appropriate boundaries between researchers and participants. Widespread replication of this method would benefit from a broad discussion among researchers, social media users, social media companies, and experts in research ethics to address appropriate protocols for such interactions.

## Introduction

As consumer health information technology (IT) becomes increasingly integral to the health care delivery system, it is imperative that such technology aligns with patients’ needs [1-4]. Gaining in-depth understanding of these requirements necessitates directly assessing patients’ existing health management practices [5,6] and experiences with prototypes of and currently available consumer health IT solutions [2,7]. Yet, obtaining in-person access to patient populations can be challenging, and researchers’ and designers’ recruitment efforts are often limited to geographically available populations due to time and cost. Consequently, research questions must fit available populations, and recruitment efforts may fail to attain populations with targeted characteristics related to race, ethnicity, and socioeconomic status. Due to their size and diversity of users, online social media platforms offer the potential to engage a wider range of patient populations in consumer health IT research.

This paper’s purpose is to present the development and assessment of recruitment strategies leveraging one social media platform, Facebook, for consumer health IT research and design. Facebook enables users to create their own profile pages and connect with others [8,9]. It also enables multiple modes of communication between individuals. Public modes include updating one’s status on one’s own profile page, posting content on another’s profile page, and commenting on others’ statuses, posts, or photos. Private modes include messaging (similar to emailing), chatting (similar to instant messaging), and video chatting (similar to video conferencing). In addition, users may create, join, and interact with groups focusing on specific topics and like, post to, and follow pages (public profiles for businesses and organizations). Each user’s home page features a news feed, a tailored selection of updates about the user’s friends’ Facebook activity [10]. Users may modify privacy settings, including specifying with whom information may be shared and from whom contact requests may be received.

Facebook is a promising recruitment instrument for three reasons. First, it remains the most visited social networking site within the United States via computer, mobile phone apps, and mobile phone browsers, with a significantly larger user base and per person usage time than the next largest social networking site across each platform [11]. Facebook reported a total of 1.28 billion active monthly users at the end of the first quarter of 2014 [12], with 71% of online adults in the United States using this service [13]. Moreover, 81.2% of daily active users reside outside the United States and Canada [14]. Consequently, Facebook may facilitate access to an extensive group of potential participants both within the United States and globally.

Second, within the United States, Facebook boasts a diversity of users [13]. No statistically significant differences exist between the proportions of online adult non-Hispanic whites, non-Hispanic blacks, and Hispanics using Facebook. Similarly, no statistically significant differences exist between the proportions residing in urban, rural, and suburban settings. Although statistically significant differences do exist across socioeconomic status and education, the largest spread between categories does not exceed 8%. The most meaningful differences in usage exist across gender and age; online women and younger adults are more likely to use Facebook. However, across both genders and all age groups except for those over 65, a majority of individuals engage with the platform. While only 45% of those over 65 currently use Facebook, this demographic is one of the fastest growing user groups [15]. Thus, directing recruiting through Facebook has the potential to engage individuals from all demographic categories.

Finally, Facebook is becoming a space for health-related activity and exchange. In 2011, Pew Research Center surveys indicated that 11% of Facebook users had posted comments, queries, or information about health or medical matters, and 9% had started or joined a health-related group on a social networking site [16]. In the same year, one study found over 600 breast cancer groups on Facebook, with a collective membership of over one million [17]. Health-related Facebook groups are used for sharing personal clinical information, requesting disease-specific guidance, receiving emotional support, fundraising, and generating awareness about a condition [17,18]. Additionally, health care organizations, including governmental centers and agencies, health care institutions, pharmaceutical companies, and nonprofits use Facebook to disseminate health advice and promotion messages [19]. Users also communicate about health through their individual profiles [20-23]. Facebook has naturally extended to encompass health-related topics; therefore, using it as a recruitment platform for health IT-related research activities is consistent with its existing scope.

Nonetheless, recruiting research participants via Facebook involves significant and novel obstacles. Encouraging people to engage in research requires more than an invitation to participate. Dillman et al have argued that participation is generated through a successfully negotiated process of social exchange, using techniques they call the “tailored design method” [24]. They note that “Tailored design is the development of survey procedures that work together to form the survey request and motivate various types of people to respond to the survey by establishing trust and increasing the perceived benefits of completing the survey while decreasing the expected costs of participation” p. 38 [24]. Creating trust and transparency is key to recruitment; however, this can be challenging in any computer-mediated communication, including group interaction. Rules and restrictions enforced by Facebook, as well as informal, emergent norms in online communities, restrict the forms and channels of communication available to researchers. As columnist David Brooks recently suggested, social media may be changing implicit assumptions about which people and which institutions are worthy of trust [25].

Within the health sciences, recruitment efforts for research via Facebook have predominantly used paid advertisements [26-32]. Clients of Facebook’s advertisement services are able to target individuals based on location, interests/hobbies, and information obtained from users’ profiles [33]. This has met with mixed results. Some studies have concluded that recruitment through Facebook advertisements is effective in terms of cost, participant yield, and ability to engage specific demographic groups such as low-income individuals and individuals with specific health conditions [27-30]. However, others have determined that this method yields few participants, or yields them at substantial cost [26,31,32], in some cases resulting in no meaningful participation [31]. This range of outcomes may be partly explained by the target populations, the subject matter of the research, and the actions requested by the researchers (eg, online survey, participation in a clinical trial). All of these variables can affect respondents’ perceptions of trust, legitimacy, benefit, and cost and determine their rate of response [24].

In some cases, therefore, advertisements are effective for recruiting via Facebook. However, the advertisement-based approach only partially capitalizes on Facebook as a platform, as it relies on broadcast strategies. Methods leveraging the social structures embedded within Facebook may provide additional means of recruiting. A few studies in the health sciences have initiated exploration of such methods. Zaid et al posted a link to an online survey to one Facebook group for women with neuroendocrine carcinoma of the cervix [34]. They received 57 survey responses within the 30-day period during which the study was open. Others succeeded by directly communicating from a study or personal Facebook page [32,35]. One study mentioned posting directly to Facebook groups and pages related to the health condition of interest but provided no details about this method or its success [23]. No health science studies we know of have used recruitment strategies that involve directly contacting administrators of multiple groups and pages or creating a study group as a sampling frame.

This paper reports on our experiences implementing two recruitment strategies to leverage the tailored design method by directly communicating with administrators and members of Facebook groups and pages. We specifically address the feasibility, benefits, and challenges of implementing such strategies within the context of consumer health IT research. Additionally, we report on our experiences targeting individuals (1) from a specific ethnic group (ie, Filipino-Americans) and (2) with a specific diagnosis (ie, type 2 diabetes) from multiple demographic groups. To our knowledge, this is the first assessment of such recruitment strategies capitalizing on the complex social structures of an online social network for health-related research.

## Methods

### Study Details

#### Study 1: Consumer Health Information Technology in a Filipino Community

Study 1 was designed to assess the feasibility, benefits, and challenges of using Facebook to recruit members of a specific ethnic group, Filipino-Americans, for a survey assessing the perceptions, use of, and preferences for consumer health IT. Recruitment occurred between August and December 2013. This study focused on Filipino-Americans because, despite Asian Americans’ extensive online presence [36], the needs and preferences for consumer health IT of specific communities within this population remain largely uncharacterized. Participants completed an online survey administered through SurveyMonkey [37], which contained 33 closed and 4 open-ended questions on topics including (1) experiences with two forms of consumer health IT (ie, personal health records and mobile health applications) and general social networking sites for health management, (2) preferences for the design of consumer health IT targeting the Filipino-American community, and (3) demographics. Eligible individuals were 18 years or older, identified as Filipino, and lived in the United States. Prior to launch, the online survey was piloted and revised based on feedback from 8 individuals identifying as Filipino-American.

#### Study 2: Informing Consumer Health Information Technology Design: How Patients Use Social Networking Sites

Study 2 was designed to assess the feasibility, benefits, and challenges of using Facebook to recruit individuals with type 2 diabetes from multiple demographic groups into a multiphased, mixed methods investigation of how patients use online social networking sites to communicate health information. This ongoing study consists of three phases: (1) qualitative exploration of participants’ health information communication practices (target N=36), (2) development and pilot of a survey instrument based on Phase 1 findings (target N=24), and (3) a large sample survey of patients’ health information communication practices (target N=600). This study’s findings will provide design guidance for consumer health IT supporting health information communication with patients’ social network members. As a first step, participants were asked to join our study’s Facebook group and complete a 23-question pre-study survey administered online through Qualtrics [38] assessing eligibility (ie, over 18, US citizen or residing in the United States, diagnosed with type 2 diabetes, Facebook user), demographics, Facebook use, interest in study phases, and preferred contact information. We explicitly asked about Facebook use in case participants had forwarded the survey link to individuals who were not yet members of our Facebook group. This pre-study survey primarily consists of questions previously used by research team members in other studies; new questions were developed based on the expertise of two research team members with extensive experience in survey methodology. Results will serve as a foundation for purposive sampling for the study phases. This paper reports on recruitment activities to acquire pre-study surveys taken by the first 100 group members (the point at which maximum variance sampling for Phase 1 interviews was initiated). These activities occurred between May and June 2014.

### Recruitment Procedures

#### Overview

Given the dearth of health sciences studies leveraging Facebook’s social structures for recruitment, we sought guidance from sociological methodology. Specifically, our strategies were informed by Bhutta [39]. Bhutta’s method involved reaching out to her own social network and to administrators of existing Facebook groups for baptized Catholics in the United States (Bhutta’s target population). Interested individuals were directed to a Facebook group created for the study, and upon joining, were sent direct messages containing a link to the study survey.

Our strategies differed from Bhutta’s in key ways. First, we reached out not only to Facebook groups, but also to pages. Second, Study 1 did not involve a study group; rather, the survey link was directly posted to relevant groups and pages. Third, in Study 2, group members were informed that the pre-study survey was an initial step in a more complex study and that they would be asked to engage in additional study-related activities such as online interviews or focus groups. Finally, to minimize potential for coercion, we did not directly advertise our study to members of our personal social networks. Below, we detail the recruitment strategy for each study.

#### Study 1: Consumer Health Information Technology in a Filipino Community

The methodology we developed and tested for Study 1 consisted of three parts: (1) identifying Facebook groups and pages targeting Filipino-Americans, (2) announcing our study to administrators of identified groups and pages (ie, gatekeepers to our target population), and (3) posting a survey link directly to consenting groups and pages. Groups and pages were identified through a keyword search of the terms “Filipino America” and “Filipino USA”. Pages or groups were excluded if they did not appear to be permanently based in the United States, if their members or followers did not appear to live in the United States, if their Facebook presence was inactive, if they appeared not to focus on the Filipino-American community, or if they did not allow others to initiate contact. Through this keyword search and application of exclusion criteria, we identified a total of 78 groups and 69 pages.

**Figure 1:**
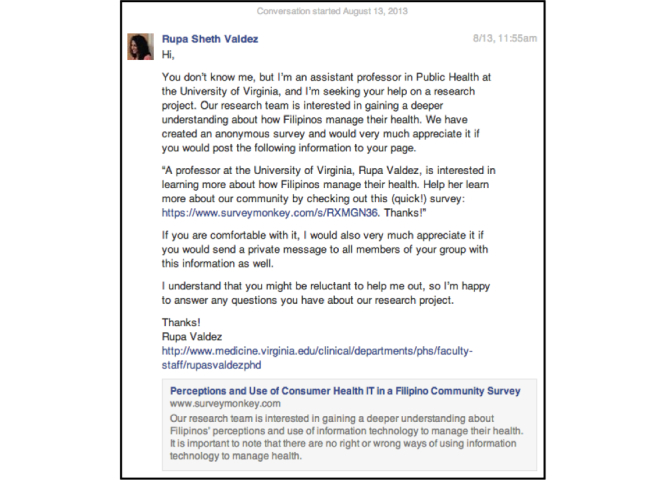
Message to Filipino-American group and page administrators.

**Figure 2:**
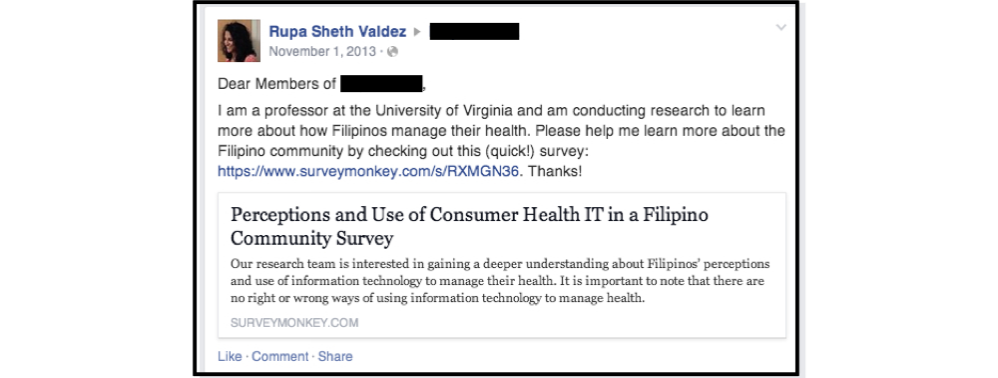
Post to Filipino-American groups and pages.

Administrators were contacted through Facebook’s private messaging function (Figure 1). In this study, we did not pay to ensure that private messages were sent to recipients’ inboxes. Consequently, some messages may have been delivered to “Other” boxes, adopted by some users to receive messages from individuals who are not among their Facebook friends. Responses were received from one group and seven page administrators. Given the low response rate, the strategy was updated to directly post our survey link to the groups and pages. Since only group members are allowed to post, we asked to join each identified group. For “Open” groups, which contain the most relaxed privacy features, membership was granted automatically. For “Closed” groups, with more extensive privacy features, permission from an administrator was required. Depending on a page’s privacy settings, posts were either automatically approved or sent to the administrator for approval. In all groups and pages for which access was granted, we posted a study announcement and SurveyMonkey link (Figure 2) a total of five times over a 4-month period.

#### Study 2: Informing Consumer Health Information Technology Design: How Patients Use Social Networking Sites

The methodology developed and tested in this study required (1) creating a study group, (2) identifying Facebook groups and pages relevant to individuals with type 2 diabetes and racial and ethnic minorities, (3) messaging group and page administrators about our study, (4) managing the study group, and (5) messaging study group members about the pre-study survey.

As a “home” for our study on Facebook, we created a group titled, “Diabetes Management Study Community”. To promote rapport, we used an image of the research team engaging in health management activities as a cover photo. We used the “About” section, visible to all Facebook users, to establish transparency of research activities. This section contained a welcome message, study description, eligibility criteria, current study activities, group rules, contact information, and information about sponsorship and institutional review board approval. Moreover, we posted files including detailed descriptions of the study and its eligibility criteria, and a list of anticipated frequently asked questions to the group’s page.

We searched Facebook to identify groups and pages for individuals with type 2 diabetes (or diabetes more generally) and for racial and ethnic minorities to promote diversity in our sample. We used keywords such as “Type 2 diabetes”, “diabetes”, “Hispanic”, “Asian American”, “Pacific Islander”, “African American”, “Chinese American”, and “Pakistani American”. It is important to note that this keyword search was not exhaustive of all potential search terms. Groups and pages were excluded if they contained fewer than 10 members or had fewer than 10 likes, their members did not appear to live in the United States, they had been inactive for the past 6 months, or they did not allow others to initiate contact. After this keyword search and application of exclusion criteria, we contacted 122 groups and 132 pages. Additionally, we contacted 1 group and 5 pages based on referrals from those previously contacted. A breakdown by target population is shown in Table 1.

**Table 1:** Study 2 group and page breakdown.

Target population	Groups contacted, n	Groups responding, n (%)	Pages contacted, n	Pages responding, n (%)
Type 2 diabetes	49	24 (49)	54	18 (33)
American Indian/Alaska Native	14	6 (43)	17	6 (35)
Asian	18	7 (39)	27	6 (22)
Native Hawaiian or other Pacific Islander	14	6 (43)	13	5 (39)
Black or African American	16	6 (38)	11	2 (18)
Hispanic/Latino	12	7 (58)	15	2 (13)
Total racial/ethnic	74	32 (43)	83	21 (25)
Total	123	56 (46)	137	39 (29)

**Figure 3:**
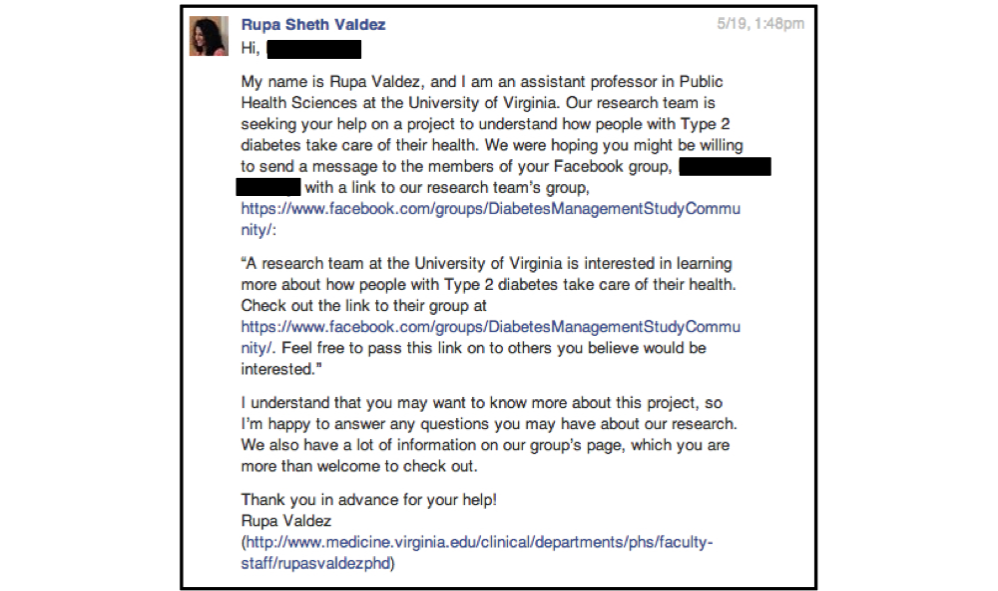
Message to groups and pages for Study 2.

Facebook’s private messaging function was used to contact administrators of each identified group and page (Figure 3). Given our low response rate from administrators in Study 1, when required, we paid Facebook a nominal fee (between US $0.19 and $1.06) to ensure that messages were routed to people’s “Inbox” as opposed to “Other” box. We paid this fee for 91 of the 123 group administrators contacted. No fee was required to send messages to pages. Follow-up messages were sent to all administrators who had not responded within 5 days. In total, administrators of 56 groups and 39 pages responded. We crafted personalized replies to each to establish trust and transparency.

**Figure 4:**
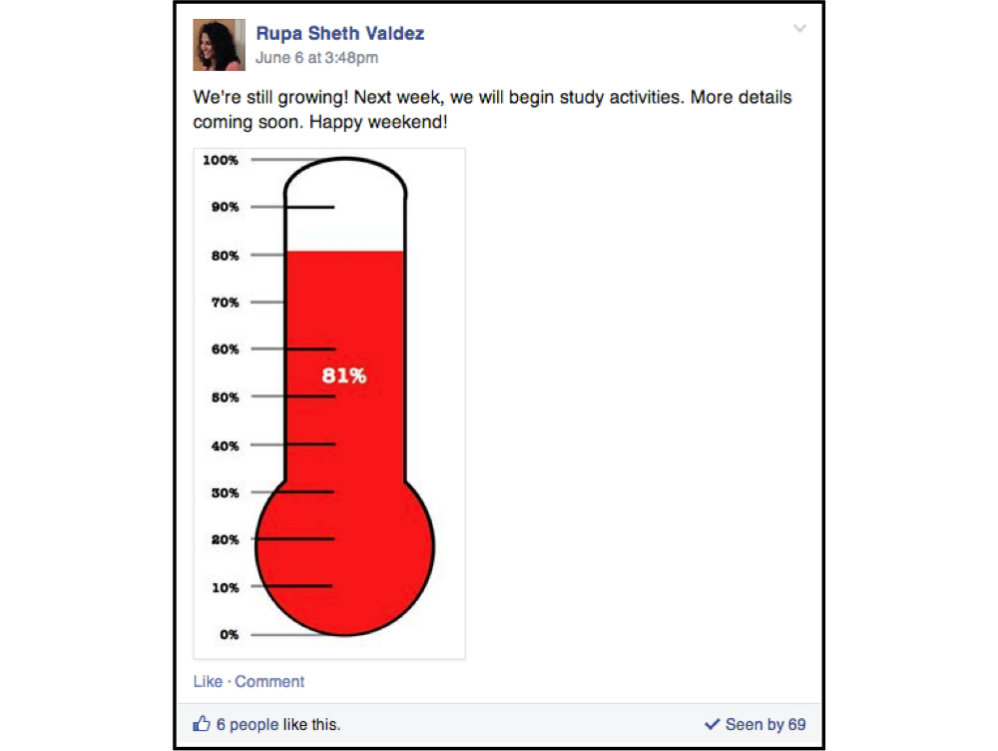
Example message posted to study group by research team.

The study group was managed to promote engagement without overwhelming members with excessive content. The research team posted weekly, informing members about study progress, encouraging them to recruit others (Figure 4) and responding to all questions that were not answered by other group members. All member-generated posts required approval by the research team. We requested that group members use the group’s page only to ask general questions and send questions about specific situations directly to the group’s moderator. Posts containing medical advice and solicitations were not approved. Initially, the research team intended to post diabetes-specific informational resources consistent with those approved by the National Diabetes Education Program (NDEP), American Diabetes Association (ADA), or other similar institutions. An initial post by the research team, however, was met with multiple comments expressing concerns because the members didn’t believe in “mainstream” guidance, but instead followed alternative methods of disease management. For the remainder of the study, the research team also adhered to the guideline of not posting messages containing medical advice.

Once our study group reached 100 members, we sent private messages to each member thanking them for joining and asking them to complete the pre-study survey. Two reminder messages were sent to all who did not complete it.

### Ethical Considerations

Both studies were approved by the University of Virginia’s Social and Behavioral Sciences Institutional Review Board. The first screen of each survey provided details about the study’s purpose and participants’ rights. Advancement past the initial screen was interpreted as provision of informed consent. No incentive for survey completion was provided in either study. However, participants completing the pre-study survey for Study 2 were informed that incentives would be provided if they were recruited into Phase 1 or Phase 2.

Participant confidentiality was maintained throughout both studies. In Study 1, survey data were collected anonymously. In Study 2, survey data were not anonymous because they were used as a platform for maximum variance sampling; however, all data were securely stored on password-protected computers and files. To further ensure privacy and security of participants’ information, we created a closed Facebook group, in which group membership is public, but only members can see posts and access group files. Additionally, we communicated with members about privacy and security issues to increase awareness. For example, in the “About” section, we reminded participants that their Facebook friends might be able to see what groups they joined and provided the option of communicating with us outside Facebook.

### Data Analysis

All survey data were exported into SPSS 21.0 [40] for analysis. The percentage of missing data and the demographic compositions of both samples were determined using descriptive statistics. All posts and messages received from group and page administrators and study group members were loaded into QSR NVivo 10 [41] for analysis. Qualitative data from both studies were analyzed concurrently using qualitative content analysis methods [42-45] that stop at a description of the data in everyday language (as opposed to theory development). The unit of analysis was the message, and simultaneous coding was used when units of text contained several meanings [46]. To ensure rigor, 3 investigators collaboratively coded the data. One investigator (MJT) created an initial coding framework for Study 1 and another (HKM) created an initial coding framework for Study 2. Both met together with a third (RSV) to create a revised coding framework encompassing both studies. The initial 2 investigators then recoded their respective datasets as appropriate, met again with the third investigator, and conducted a final review of the other investigator’s dataset to ensure consistency.

## Results

### Recruitment

#### Study 1: Consumer Health Information Technology in a Filipino Community

**Figure 5:**
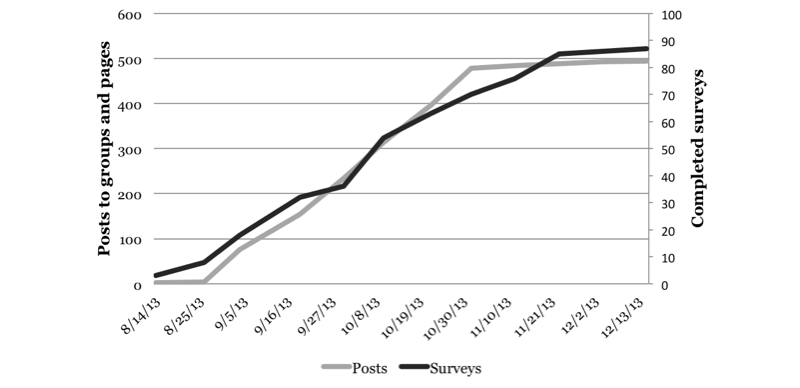
Study 1 cumulative recruitment over time.

We successfully posted our study message to 31 of the 78 identified Facebook groups (40%) and 62 of the 69 identified Facebook pages (90%). During the study, our posts were “seen” a total of 3564 times. Facebook indicates that a post has been seen by anyone who scrolls past or follows a link directly to a post. Consequently, someone who “sees” a post may not necessarily read it. Our posts to groups received 65 “likes”, and our posts to pages received 9 “likes”. Additionally, our posts were shared three times, and the principal investigator received 9 friend requests from members of the target population. The survey was accessed 137 times of which 87 resulted in completed surveys. The rate of study completion was proportionate to that of posting to groups and pages (Figure 5). Approximately 6% of survey data were missing for the demographic variables and 2% for the remaining 22 close-ended variables; 91% of the respondents provided substantive feedback on at least one, 86% on at least two, and 75% on at least three of the four open-ended questions. No direct costs beyond staff labor were incurred.

#### Study 2: Informing Consumer Health Information Technology Design: How Patients Use Social Networking Sites

**Figure 6:**
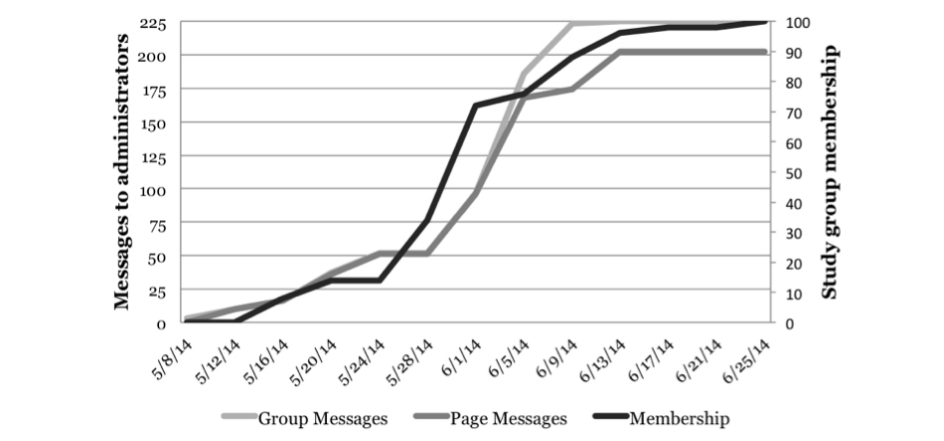
Study 2 cumulative Facebook study group membership over time.

We received replies from administrators of 56 of the 123 contacted groups (45%) and 39 of the 137 contacted pages (28%). Table 1 shows a breakdown by type of group and page. Eight administrators volunteered to share information about our study via Twitter, blogs, newsletters, listservs, or Google groups. Because we were not members of the groups, we were unable to determine how many Facebook users had “seen” or “liked” posts containing our study information. The principal investigator received friend requests from 3 administrators and 1 group member. Figure 6 displays numbers of messages sent to group and page administrators and membership of our study group. Membership growth seems to have followed not from the numbers of messages but rather the actions of a subset of administrators. From the 100 group members, we received 79 completed pre-study survey responses from unique individuals (after checking for duplicate Internet protocol [IP] addresses and contact information), of which 61 were from eligible individuals. Approximately 1% of survey data were missing. Total direct costs incurred (excluding investigator and staff effort) totaled US $118.17, equaling $1.94 per completed survey from eligible individuals.

### Participant Characteristics

#### Study 1: Consumer Health Information Technology in a Filipino Community

Demographic characteristics of the 87 respondents who completed the survey appear in Table 2. Gender was evenly distributed. All age and income brackets were represented, with the highest number of participants between ages 30 and 49 and with a household income between US $30,000 and $74,999. Participants were more likely to be married, speak English at home as a primary language, and have at least an Associate’s or Bachelor’s degree. Slightly more individuals reported the Philippines, rather than the United States, as their country of birth; 93% strongly or very strongly identified as Filipino.

**Table 2:** Study 1 demographic characteristics (n=87).

General characteristics		n (valid %)
**Gender**		
	Male	40 (49)
	Female	41 (51)
**Age**		
	18-29	18 (22)
	30-49	26 (32)
	50-64	24 (29)
	65+	14 (17)
**Education**		
	Less than high school	1 (1)
	High school diploma or equivalent	2 (2)
	Some college, but no degree	11 (13)
	Associate’s or Bachelor’s degree	35 (42)
	Graduate degree	34 (41)
**Household income, US$**		
	<$30,000	17 (22)
	$30,000-$74,999	26 (34)
	$75,000-$99,999	13 (17)
	$100,000+	20 (26)
**Marital status**		
	Married	48 (59)
	Widowed	7 (9)
	Divorced	4 (5)
	Separated	1 (1)
	Never married	22 (27)
**Birth country**		
	Philippines	46 (55)
	United States	36 (43)
	Other	2 (2)
**Primary language at home**		
	English	61 (75)
	Tagalog	13 (16)
	Other	6 (9)
**Identify as Filipino**		
	Very strongly	59 (70)
	Strongly	19 (23)
	Neutral	5 (6)
	Not at all strongly	1 (1)

#### Study 2: Informing Consumer Health Information Technology Design: How Patients Use Social Networking Sites

**Figure 7:**
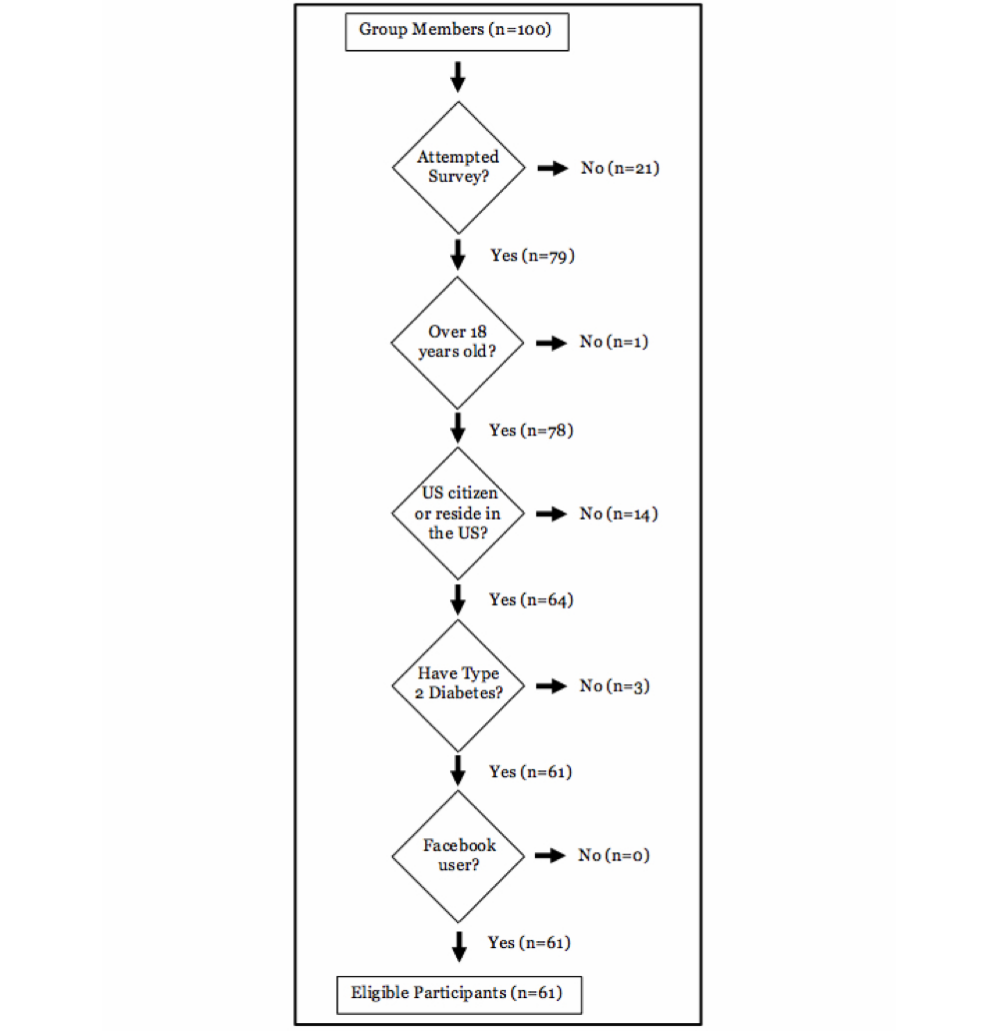
Reasons for Study 2 ineligibility.

The pre-study survey was completed a total of 79 times, excluding known duplicate attempts, for a response rate of 79%. However, 18 individuals were deemed ineligible (Figure 7). Of the 61 eligible individuals (Table 3), the majority were female, between the ages of 30 and 64, and married. All but one had a high school diploma or equivalent and 76% had at least some college education. All geographic and household income categories were represented, although only two participants reported a household income over $150,000. Eligible individuals completing the pre-study survey were predominantly white, with moderate participation from the black/African-American and Hispanic/Latino communities. Most indicated interest in more than one study phase.

**Table 3:** Study 2 demographic characteristics (eligible only) (n=61).

General characteristics		n (valid %)
**Gender**		
	Male	15 (25)
	Female	46 (75)
**Age**		
	18-29	1 (12)
	30-49	22 (36)
	50-64	29 (48)
	65+	9 (15)
**Education**		
	Less than high school	1 (2)
	High school diploma or equivalent	11 (18)
	Some college, but no degree	21 (34)
	Associate’s or Bachelor’s degree	18 (30)
	Graduate degree	10 (16)
**Household income, US$**		
	<$30,000	20 (33)
	$30,000-$74,999	22 (36)
	$75,000-$149,999	17 (28)
	$150,000+	2 (3)
**Marital status**		
	Married	42 (69)
	Widowed	2 (3)
	Divorced	11 (18)
	Separated	2 (3)
	Never married	4 (7)
**Geographic region**		
	Urban	14 (23)
	Suburban	20 (33)
	Rural	23 (38)
	Other	2 (3)
	Don’t know	2 (3)
**Race**		
	White	45 (77)
	Black or African American	7 (12)
	American Indian/Alaskan Native	1 (2)
	Asian	1 (2)
	Native Hawaiian/Pacific Islander	0 (0)
	Some other race	4 (7)
**Ethnicity**		
	Hispanic or Latino	6 (10)
	Not Hispanic or Latino	55 (90)
**Interest in study phases**		
	Phase 1	43 (70)
	Phase 2	40 (66)
	Phase 3	48 (79)

### Content of Interaction

#### Study 1: Consumer Health Information Technology in a Filipino Community

We received feedback via 21 messages and 27 comments during recruitment, reflecting seven broad themes. Individuals noted their interest in providing assistance with our recruitment, confirming participation, declining participation, and providing referrals to other organizations for recruitment. They also requested clarification of multiple dimensions of our study, including our purpose, methods, and target population. A few voiced concerns. For example, one administrator warned fellow group members about the security of their information; another expressed unease at the number of times we asked to post the recruitment message. Others provided encouragement for our research through emotionally supportive messages and instrumentally supportive messages seeking to connect us with other individuals and organizations to partner with in the future. We also received messages expressing interest in our results and us as individuals (eg, requests to be Facebook friends). Some individuals offered promotion of our study to others. Finally, we received a few messages containing solicitations. Verbatim examples are shown in Table 4.

**Table 4:** Study 1 messages and comments received.

Theme	Study 1 message and comment quotes
Assistance	“Hi Rupa, Thank you for reaching out to the [name of organization] facebook page for your research study. I would recommend going to our website at [url]. We have a listing of all the Filipino Organizations that belong to [name of organization] (it is an umbrella organization) with emails and contacts that may be of help to you. I unfortunately cannot post your post because our facebook page is reserved for paid partner organizations events and promotions only. I hope you find the information above helpful and good luck with your study! Sincerely, [Name] [Job title]”
	“I am Pastor of [name] Church and President of [name of organization] of [region]. I have checked out your web page at UVA and am willing to help. I will post this in both pages. Hope this helps.”
Clarification	“where/how are you publishing results?”
	“By the way, why did you choose to choose Filipino Community as the overall subject of this research?”
Concerns	“I took this survey and it has been verified as safe. No personal information will be requested. Thank you professor for your interest. For those interested using social media and other internet based applications to discuss personal health issues; be careful and use common sense. The Internet (wireless in particular) is an unsecure means of communication. Putting information about your health online may effect things such as employment and insurance.”
	“You have submitted this 4 times already…”
Encouragement	“Ms. Valdez, You may want to try to partner with direct service agencies who service Filipinos. Two of them are [name of organization] and [name of organization]. Both are located in [city] (within [county]) Good luck with the survey.”
	“Hello, Ms. Valdez. This is an interesting study/survey. The [name of organization] thanks you and we hope that you’ll get many participants and useful results to your survey and good luck!”
Interest	“No problem Rupa! I am very interested to hear about the results of your study. If you are able to would you be able to email me your findings when completed?”
	“I am the Pastor of [name of Church]. Please add me as a friend. Thank you! [url]”
Promotion	“Simple survey to fill out. Takes about 5-10 minutes. (Pork adobo, sisig, and chicharron...hmmm good)”
	“I encourage more people to take this survey. I already manage my healthcare online through my HMO’s website. I email my doctor and can go back and view the discussion anytime. I schedule and cancel appointments with ease online. I check pharmacies to see if my medications are available then order them and pick them up. I can view my medical test results anytime I want online. Also a medical chart ID app is like an easy access medical identification which is most valuable in case of emergencies and crisis were split second decisions are made that can save your life.”
Solicitations	“Hello po kabayan, I would like to share to you a very promising pinoy product. [name of product] - a combination of wheatgrass and guyabano. Be a dealer now and get all [name of product] products half the price (50% lifetime discount upon membership). See more of the privileges as u become part of our team! [url]”
	“Please help these families in need [url]”

#### Study 2: Informing Consumer Health Information Technology Design: How Patients Use Social Networking Sites

We received 92 messages from administrators and study group members while recruiting. These reflected six of the themes seen in Study 1—*assistance, clarification, concerns, encouragement, interest, and solicitations*—and one new theme, *health information*. Individuals commented on their ability to provide recruitment assistance; however, the scope of these messages was larger than in Study 1. Specifically, we received messages stating that individuals needed time to consider participation and providing reasons for declining participation, such as language barriers or inconsistency with the group’s privacy guidelines or scope. Concerns were expressed regarding whether or not we advocated the ADA’s views of diabetes management, the historical treatment of minority populations in scientific research, and the perceived vagueness of our study description. Individuals indicated interest in participating in future studies, but not in study findings or the research team. Messages related to clarification, encouragement, and solicitations paralleled those received in Study 1. A few individuals sent us messages containing health information specific to their situation. Verbatim examples are shown in Table 5.

**Table 5:** Study 2 messages received.

Theme	Study 2 message quotes
Assistance	“Hi Rupa, we thank you for reaching out and thinking of us for this cause. Unfortunately, we are unable to share your link on our page due to our community guidelines. We wish you the best of luck and thank you for your commitment toward bettering the lives of those affected by diabetes.”
	“I will join the group myself and see what it is about and then I will decide if I wish to pass the information on to my other group members.”
Clarification	“What will the information you gather go towards? New guidelines for type 2?”
	“Are you seeking this from the Arab American community or anyone can participate?”
Concerns	“I need to know whether your group is going to listen to concepts that may run contrary to the ADA. Many of us believe that the ADA is very narrow-minded and actually has caused more damage than helped. Need to know where you stand with traditional concepts.”
	“I am an Indian advocate and it has been our cultural that these studies are never good for our culture. With broken treaties, feeding our relatives alcohol, bad government food in the name of the commodities program, isolation on the most desolate reservations and colonies. Fake blood tests that were really designed to get our DNA, trust is an issue for natives.”
Encouragement	“Ma'am, I am not an expert on groups, but I would like to see if you would like to observe of how we run our group from the admin side. This will give you important insight on what we have found to work and what did not, how we communicate with each other, and how we run our group as tightly with less spam that most. Let me know if you would want to do this. I think it might help you to not make many of the mistakes that we did starting out.”
	“Hi Rupa, Of course I will share the message! Sorry it took so long to get back to you. I was on vacation for the past two weeks and haven’t done a ton of updating. Good luck in your study! Posted! J”
Health Information	“I have changed my eating habits and eat healthy foods now. I eat fish and chicken baked, veggies and fruits (berries, apples and citrus) occasionally bananas for the potassium. I drink a gallon of water a day and walk when possible. I take vitamin b complex, vitamin c and a multiple vitamin daily. I hope this information helps. Losing weight has helped as well. I am still working on losing more but the 40 pounds I have lost has helped a lot.”
	“I have type 2 diabitees lamtaking insulin twice aday”
Interest	“If you have any studies on type 1 please let me know, I would love to participate. I know [name] who works there at UVA in the [name] dept. She is a [position] with your university. I just live too far away to participate in the [name] project stuff unfortunately.”
	“Thanks for the return. It is ok thanks. Well if you do get the ok to go global please keep me in your records. My wife is diabetic too by the way. May it go well and you get the results that you are searching for. Kindest regards and thanks for conscidering me even if unsuccessful this time round. Sincerely”
Solicitations	“In return could I ask that you take a look at the real diabetic reader reviews page for my book [name] [url] and send the link to people you know with Type 1 and Type 2 diabetes.”
	“Hi Rupa, So sorry for the lag time. I have been on vacation and trying to get through everything. Yes, we would be happy to post this on our Facebook page. I will plan to post it tonight. I would like to ask if you would be willing to post about [page name] on your Facebook page. If so, please let me know, and I will send a post over to you. Thank you!”

Study group members contributed 26 posts and 14 comments to our group’s page, not including messages that remained unapproved because they violated our community’s guidelines (eg, spam, medical advice). These posts and comments reflected three themes. Members expressed appreciation for the study and for acceptance into the Facebook group. Upon acceptance, many shared health information, including personal and family medical histories. Others asked for and provided emotional, technical, and instrumental support to fellow community members. Verbatim examples appear in Table 6.

**Table 6:** Study 2 posts and comments on study group’s Facebook page.

Theme	Study 2 quotes of posts and comments on our Facebook group’s page
Appreciation	“Thank you for adding me to this group! I am a type 2 diabetic and have been since i was 16.”
	“Thank you for accepting me into the group. I am a T2 for about 20 years. Have been on a pump since Dec 2009. It has changed my life. Looking forward to the study”
Health Information	“Diagnosed in 1992. Strong family history on my fathers side. On OmniPod since 2012. I love my pod and am convinced pumps are best. Currently in a bad slump of diabetes burn out.”
	“Interesting to see where this leads. Probably borderline for 20 years.. Mild T2. Controlled by diet and exercise. Side benefit of retirement - time to exercise regularly. A1C 6.1”
Support	“I took the survey but haven’t heard anything. I am not sure how to get to the site only got here because I got a notification! Can you tell me what to do?”
	“[name], go to Notifications on the top right and click to turn off notifications from any group you are a member of.”

## Discussion

### Principal Results

The Study 1 recruitment method yielded 87 complete survey responses, and the Study 2 method 79 complete (61 eligible) pre-study survey responses. The first method yielded a completion rate proportionate to that of the rate of posts made, whereas successes of the second method seemed to follow directly from the actions of a subset of administrators. Direct costs, excluding personnel time, were negligible, with none incurred in Study 1 and US $118.17 in Study 2. In implementing these recruitment strategies, we received messages, posts, and comments reflecting 10 themes: appreciation, assistance, clarification, concerns, encouragement, health information, interest, promotion, solicitations, and support.

### Feasibility

Leveraging Facebook for recruitment was more successful for obtaining small samples for qualitative research than large samples for quantitative research. With 87 and 61 complete responses from eligible participants in Study 1 and Study 2 respectively, our strategies did not yield sufficient participation for conducting large sample surveys, in contrast to the results obtained by Bhutta [39], whose recruitment strategies served as a basis for our methodologies. After contacting 42 groups in a 1-month period, Bhutta obtained over 4000 completed surveys from baptized Catholics in the United States. It is unclear to what this difference in sample size may be attributed. Although the prevalence of Catholicism (24.3%) [47] in the United States is greater than the prevalence of diagnosed diabetes (8.3%) [48] and the proportion of Filipinos (0.8%) [49], this difference may explain the recruitment disparity for Study 1 but is not large enough to account for the disparity in Study 2. As Bhutta conducted her study in 2008, another factor may be changing attitudes regarding privacy on Facebook and subsequent changes in use patterns [50,51]. In addition, unlike Bhutta, our methodologies did not use our personal Facebook networks for recruitment. Bhutta does not report on how participants learned about her study; however, the act of recruiting friends or friends of friends who may have greater trust in the research may have meaningfully contributed to her study’s successful recruitment outcomes.

Given the wide range of recruitment results demonstrated by researchers using Facebook advertisements, it is difficult to ascertain the effectiveness of our method in relation to this alternative. Using advertisements, health sciences investigators have reported as many as 1548 [29] and as few as zero [31] completed surveys. In terms of sample size, ours were closest to that of Lohse [27], who reported under 100 participants for each of two advertising campaigns related to nutrition. In contrast, our direct costs were substantially less than those incurred by all Facebook-based advertisement campaigns. Whereas as our strategies resulted in no direct cost (Study 1) or a cost of $1.94 per eligible participant (Study 2), others have reported direct costs (not including personnel time) ranging from $4.28 [29] to $32.26 [27] per participant. Studies using Facebook advertising campaigns have reported a range of personnel time required for executing the strategy [29,32]. Our strategies required a fair amount of effort to identify Facebook groups and pages, generate personalized responses to inquiries, and moderate the study group. However, a significant portion was devoted to specifying protocols for each task. Consequently, we believe the time required to execute each strategy would be significantly reduced upon subsequent applications.

Both strategies demonstrated potential in recruiting participants for qualitative inquiry. In Study 1, a large majority of participants provided substantive responses to open-ended questions. In Study 2, we were able to recruit our target of 100 group members to serve as a sampling frame for our 36 qualitative interviews. Given that previous studies using Facebook advertisement campaigns have primarily explored their potential for quantitative research, there are no published studies that we can use to assess the effectiveness of our recruitment strategies against the use of advertisements for qualitative research.

### Representativeness

#### Study 1: Consumer Health Information Technology in a Filipino Community

Comparisons between our study population and data from the 2012 American Community Survey (ACS) [49] were used to assess the representativeness of our sample. Whenever possible, comparisons were direct; however, for a subset of variables, direct comparisons were challenging given differences in data collection or absence of relevant data from the 2012 ACS. Our sample was reasonably representative in terms of gender, age, marital status, and birth country. In contrast to the general Filipino population living in the United States, our sample was more likely to be older than 65. This was unexpected, given that individuals over 65 are the least likely to be Facebook users [13] and that older Asian-Americans are less likely to use social media regularly [36]. Moreover, our sample contained a higher proportion of married individuals and a lower proportion of never married individuals than the US Filipino population. This may have resulted from the fact that our sample was overrepresentative of individuals with higher educational attainment, who are more likely to be married [52]. Assessing representativeness in terms of household income, language spoken at home, and identification as Filipino were infeasible given available data.

#### Study 2: Informing Consumer Health Information Technology Design: How Patients Use Social Networking Sites

Directly comparing our study population to the true US diabetic population on Facebook was not possible given available data. However, it is possible to estimate the proportions of the American diabetic population on Facebook within each gender and ethnic/racial category using available data and some simplifying assumptions. The estimates are based on nationwide gender and ethnic/racial population size [49], diabetes diagnosis rate for these populations [53], and estimates of Internet usage and (among Internet users) Facebook usage by these populations [13], under the simplifying assumption that gender, race/ethnicity, Facebook usage, and diabetes are independent of each other. Based on these estimates, our study was highly over representative of women, expected to comprise 51.0% of the diabetics on Facebook (women are more likely to be on Facebook, but men are more likely to have diabetes—differentials that roughly balance each other out in our calculations). To explain the gender disparity in recruitment, one would need a better understanding of how men and women use Facebook groups and pages, the basis of our recruitment. Compared with our estimated populations of diabetics on Facebook, our study was reasonably representative of whites and American Indian/Alaskan Natives, over representative of blacks/African Americans, and under representative of Hispanics/Latinos, Asians, and Native Hawaiian/Pacific Islanders. It is unclear what contributed to the underrepresentation of these latter three groups. Factors may include the activity level of our contacted Facebook groups, frequency of Facebook group use among these populations, and group differences in attitudes related to trust in scientific research. Furthermore, although we contacted groups and pages conducted in multiple languages (eg, Spanish and English), budgetary constraints limited study participation to individuals comfortable communicating in English. We have initiated efforts to strengthen racial and ethnic representativeness of our sample by contacting additional groups and pages; this has yielded moderate improvement in the numbers of individuals identifying with currently underrepresented racial and ethnic groups.

### Ethical Challenges

These studies prompted ethical deliberations. Determining whether to accept friend requests from administrators and potential or actual participants was challenging given that qualitative research requires building trust between researchers and participants while maintaining appropriate distance [54-56]. We opted not to accept friend requests, primarily due to a desire to protect the privacy of our personal lives. We considered creating alternate accounts specifically for research; however, Facebook’s Statement of Rights and Responsibilities only allows one account per user [57,58]. Another option would have been to create a Facebook page to act as our public face. We decided against this option in order to facilitate peer-to-peer relationships between members of the research team and study participants. Although we can only speculate about motivations for the friend requests, it was likely that individuals wished to confirm our legitimacy and learn more about us. Consequently, instead of accepting friend requests, we actively encouraged interaction through private messages and the study’s group page. Several members of the research team also expanded the information publicly available on their Facebook profiles.

Another ethical issue concerned messages from our targeted racial and ethnic groups and pages in Study 2. Although we received many supporting our recruitment efforts, we also received messages stating that our request was unrelated to the purpose of the contacted group or page. We approached groups or pages serving racial or ethnic communities of interest without excluding those unrelated to health (eg, film, engineering, photography, radio). By casting a wider net, we hoped to increase our recruitment of racial and ethnic minorities, ensuring that any recommendations for consumer health IT design were culturally relevant to multiple populations [59,60]. However, it is possible that we inadvertently alienated members of the populations we tried to engage. In future recruitment, we will explicitly state our reasons for targeting such groups (ie, to increase the representativeness of our sample).

### Considerations for Future Research

Leveraging Facebook for recruitment requires building rapport with individuals online. Communication with gatekeepers and potential respondents must establish legitimacy, create trust, promote transparency, and respond effectively to concerns about participation. It is unclear what benefits and challenges researchers would encounter in repeatedly implementing this strategy, particularly with the same target population. Creating online relationships with administrators could lay a foundation for community-based research in which long-term relationships are established with community members as full partners. Conversely, frequent requests by multiple researchers may result in action against perceived spam, unless interactions are established to add greater value for participants. However, creating greater value for participants is challenging in that participants may have varying beliefs about disease management and varying levels of comfort in sharing personal information. Broader engagement with research participants may also heighten the probability of ethical dilemmas. In particular, if the research team contains clinicians, it may be difficult to balance their multiple professional roles.

Evolving features within social media may further contribute to uncertainty about and the complexity of operationalizing this method over time and across researchers. Facebook’s privacy settings are continually changing [61-63]. Researchers cannot rely on the availability of specific communication pathways with individuals to whom they are not directly connected. Similarly, algorithms for determining which posts users see remain in flux [64,65] and costs for increasing the likelihood that a post or message is seen continue to expand. Addressing these issues effectively may require researchers to contract with specialized services that understand existing social media policies to craft protocols that best meet their needs. Moreover, a broad discussion among researchers, social media users, social media companies, and experts in research ethics is necessary to address appropriate protocols.

### Conclusions

The advent of social media represents a potential solution to recruitment challenges consumer health IT researchers confront. The two studies detailed here suggest that leveraging Facebook is currently a viable means of recruitment for qualitative but not large-scale quantitative research. Given that most health-related research on Facebook has recruited through advertisements, additional research is needed to determine the long-term ethical and practical implications of adopting these alternative methods.

## Abbreviations

ACS: American Community Survey
ADA: American Diabetes Association
IP: Internet protocol
IT: information technology
NDEP: National Diabetes Education Program
